# The Effect of *C*-Terminal Helix on the Stability of FF Domain Studied by Molecular Dynamics Simulation

**DOI:** 10.3390/ijms13021720

**Published:** 2012-02-07

**Authors:** Liling Zhao, Zanxia Cao, Jihua Wang

**Affiliations:** 1 Shandong Provincial Key Laboratory of Functional Macromolecular Biophysics, Dezhou University, 566 University Rd. West, Dezhou 253023, China; E-Mails: zhaoll@dzu.edu.cn (L.Z.); qiayilai@mail.ustc.edu.cn (Z.C.); 2 Department of Physics, Dezhou University, 566 University Rd. West, Dezhou 253023, China

**Keywords:** FF domain, molecular dynamics simulation, *C*-terminal helix, structural stability, intermediate state

## Abstract

To investigate the effect of *C*-terminal helix on the stability of the FF domain, we studied the native domain FF3-71 from human HYPA/FBP11 and the truncated version FF3-60 with *C*-terminal helix being deleted by molecular dynamics simulations with GROMACS package and GROMOS 43A1 force field. The results indicated that the structures of truncated version FF3-60 were evident different from those of native partner FF3-71. Compared with FF3-71, the FF3-60 lost some native contacts and exhibited some similar structural characters to those of intermediate state. The *C*-terminal helix played a major role in stabilizing the FF3-71 domain. To a certain degree, the FF domain had a tendency to form an intermediate state without the *C*-terminal helix. In our knowledge, this was the first study to examine the role of *C*-terminal helix of FF domain in detail by molecular dynamics simulations, which was useful to understand the three-state folding mechanism of the small FF domain.

## 1. Introduction

Small single-domain proteins have important roles in studying protein conformational and dynamical properties. Studying these proteins reveals the principles of protein folding. At the same time, it is useful for optimizing a predictive computational method. Many single-domain proteins are thought to be a two-state transition between folded and unfolded states and their folding is a process of cooperative nucleation-condensation [1–3]. With the increase in research concerning single-domain proteins, some have been found with three-state behaviors such as bacterial immunity protein Im7 [4], FF domains [5–7], SH3 domains [8,9], single-chain monellin [10], hUBF HMG box-5 domain [11].

**Figure 1:**
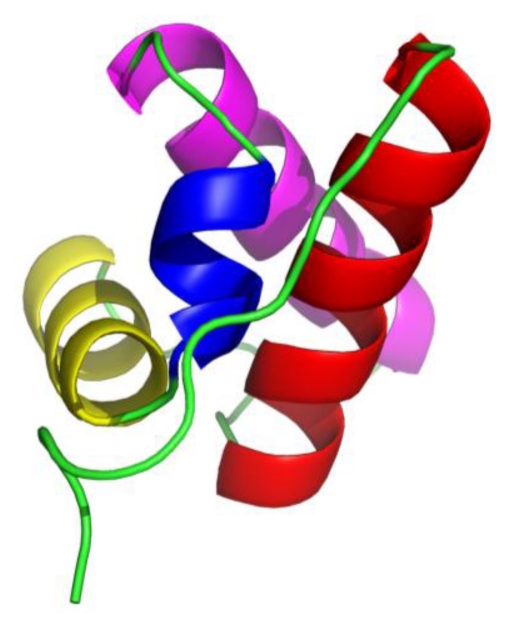
The native structure of FF domain from HYPA/FBP11 (PDB entry 1UZC, the first conformation). The four different colors were used to identify helices H1 (red), H2 (yellow), H3 (blue), and H4 (magenta).

FF domains have about 60–71 amino acid residues involved in transcription, RNA splicing, and signal transduction, and often exist in tandem arrays [12]. They present in a variety of eukaryotic proteins including the transcription factor CA150 [12,13], and the splicing factor Prp40 [14]. FF domains are small protein-protein interaction modules and their folding process includes two phases, one is a fast change from the unfolded state to intermediate state and the other is a slower, rate-limiting transition from intermediate state to the native state [7]. The native structures of FF domain from different proteins have been confirmed and deposited in Protein Data Bank (PDB) [12–15]; these are composed of four helices (from *N*-terminal marked as H1, H2, H3 and H4, respectively) arranged in orthogonal bundle, the third helix H3 is a short 3_10_ helix and the other three are α-helices (Figure 1).

FF domain from human HYPA/FBP11 is a 71 amino acid residue fragment and the native structure has been determined using standard nuclear magnetic resonance (NMR) methods [15]. This domain has been detected with an on-pathway intermediate state during the folding process by different experimental methods [5–7]. Because of short life-time and low population of the intermediate state under normal conditions, it is a difficult task to confirm and to characterize these states. Korzhnev DM and co-worker [5] studied this domain with NMR spectroscopy and found that helices H1–H3 were at least partially formed, but helix H4 was largely disordered in folding intermediate state. Jemth P. and co-worker [16] characterized the transition state between intermediate and native state by *Φ*-value analysis with 50 mutations, and declared that a folding nucleus centered at the end of H1 and the beginning of H2 came into being at this state. Dmitry M and his co-worker [17] pictured the “invisible” folding intermediate state of FF domain in detail with NMR spectroscopy and computational methods. In the intermediate state of FF domain, they found that the first two helices, H1 and H2, had a similar formation to those in the native state; the H3 was lengthened and changed into α-helix, and the H4 was badly formed. The non-native H3 prevented the formation of the native H4. The same work also indicated that the truncated FF domain with *C*-terminal helix being deleted was well folded and had a nearly identical structure to that of the folding intermediate state.

Molecular dynamics (MD) simulation is an important computational technology on biomolecular systems, which detects minute changes and ascertains the dynamical properties of the systems. With the large increments in computer power, the MD simulation has become an effective method in investigating the folding and unfolding of biomolecular systems [18–20].

In the present investigation, FF domain from human HYPA/FBP11 (FF3-71) and its truncated version with the *C*-terminal helix being deleted (FF3-60), were studied by MD simulations to determine the effect of the *C*-terminal helix on the stability of the domain. The GROMACS package and GROMOS 43A1 force field were adopted together with explicit SPC water model in the simulations. The conformational changes were assessed by parameters such as the radius of gyration, root mean square deviations of Cα atoms, native contact formation, the interactions between helices and the probability of helix formation. The results indicated that the deletion of the *C*-terminal helix induced obvious conformational transitions and the truncated version became less stable than the native domain. The truncated version displayed some similar characteristics to the intermediate state.

## 2. Results and Discussion

At first, the conformational cluster analysis [21] was carried out on the simulation trajectories of the two systems with 10 ns intervals. The RMSD value of backbone atoms was the similarity criterion with cutoff of 0.09 nm for FF3-71 and 0.1 nm for FF3-60. Only the clusters that make up 95% of a trajectory were taken into account and counted as a function of time [22]. The number of clusters did not change after 160 ns (Supplement Figure S1). Then, we calculated the distributions of radius of gyration (Rg) for two systems and found the distributions had no obvious changes during the last 160-ns trajectories (Supplement Figure S2). Finally, the conclusion could be drawn that the simulation convergence was obtained approximately. The last 160-ns simulation trajectories were taken into account for the analysis. To depict the conformational transformation, we focused on helix formation, native contact formation, root mean square deviation of Cα atoms from native state (RMSDCα), Rg, interaction of the helical interface and the spiralization or despiralization of some residues.

### 2.1. The Root Mean Square Deviation

**Figure 2:**
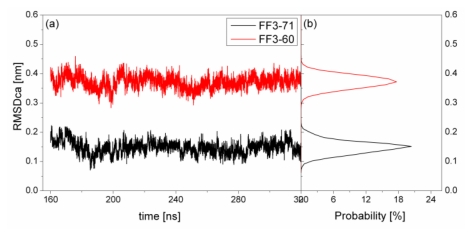
RMSDCα (residues T13 to E57) from native structure as a function of simulation time (left panel) and distributions (right panel) for native domain FF3-71(black line) and truncated version FF3-60 (red line).

To test the structure deviation from initial conformation of the simulation, RMSDCα should be considered. We calculated RMSDCα from the native structure to analyze structural transformation caused by the deletion. The RMSDCα of the residues T13 to E57 were shown in Figure 2 as a function of simulation time together with their distributions. For FF3-71, the RMSDCα changed from 0.07 nm to 0.22 nm and the value 0.15 nm had the maximal probability of 20%. In contrast, the truncated version FF3-60 had larger RMSDCα changing from 0.29 nm to 0.46 nm and the value 0.37 nm had the maximal probability of 17%. The great difference of RMSDCα of the two systems meant the large structural difference between the two systems trajectories. The central structures of the major conformational clusters of two proteins indicated that the orientation of H1,the position of H2 relative to H3 were changed, residue 51 was spiral and the length of H3 was longer than that in FF3-71 (Supplement Figure S3). That is to say, the deletion of the *C*-terminal helix brought the instability relative to the native partner FF3-71.

### 2.2. Helices Formation

**Figure 3:**
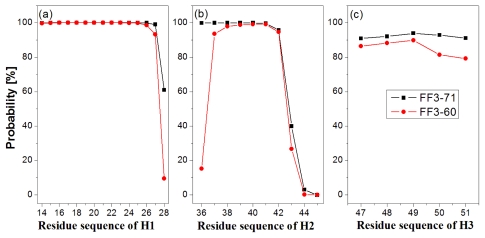
The formation of helices of two systems for residues residing in α-helix H1 (**a**), α-helix H2 (**b**) and 3_10_-helix H3 (**c**). Black square corresponds to FF3-71 and red circle corresponds to FF3-60. The helix classification was confirmed by program STRIDE. In the native state, the helical boundaries are 14 to 28, 36 to 45 and 47 to 51 and the corresponding lengths of residues are 15, 10 and 5 for H1, H2 and H3, respectively.

In order to test the effect of the *C*-terminal helix on the formation of helices H1–H3, we calculated the probability of helical formation during the simulations. In the native state, the helical regions are 14 to 28, 36 to 45 and 47 to 51 for H1, H2 and H3 and the corresponding lengths of helix are 15, 10 and 5, respectively. The secondary structure classification was confirmed by program STRIDE [23]. The simulation trajectories were analyzed by SPRIDE and the formation of secondary structure for each residue could be obtained. The helix formations during the simulations for each residue residing in the helices H1~H3 were shown in Figure 3. The helix formation probability of residue was the ratio of the conformation number in which this residue formed helical structure to the total conformation number. We also studied helix formation probability with different length (Supplement Figure S4). The length of helix was the number of residue forming helical structure in a conformation determined by program STRIDE. For instance, the *N*-residue helix meant there were only *N* residues forming the helical structures and the other residues did not form helical structures in a certain region.

First, the helix H1 formation was discussed. The formation probability of α-helix H1 was highlighted in Figure 3a. In FF3-71 most residues of H1 retained helical structure during the simulations except the *C*-terminal residue K28 only, with a probability of 61%. H1 was completely formed with probability of 61% and formed 14-residue helix with the probability of 38% in FF3-71 (Supplement Figure S4). By comparison, in FF3-60 the residues E27 and K28 had less chance to form helical structure with probability of 93% and 10%, respectively. The native helix formation probability in FF3-60 was decreased to 10% and 14-resdiue helix was preferred with probability of 84%.

Then we took the helix H2 formation into account. The formation probability of α-helix H2 was highlighted in Figure 3(b). In FF3-71 the last three residues of C-termini had no more than 40% chance to form helical structure, H2 had no chance to form the whole native helix and had the most probability of 56% for 7-residue helix (Supplement Figure S4). Compared with those in FF3-71, the *N*-terminal residues W36 had also obvious despiralization in FF3-60. H2 was preferred 6-residue helix with 56% in FF3-60 (Supplement Figure S4).

Finally the helix H3 formation was analyzed. The formation probability of 3_10_-helix H1 was shown in Figure 3c. No residue could keep helical structure throughout the simulations. In FF3-71, the native helix H3 was completely formed with probability of 89% and had also a 6% chance to completely disappear (Supplement Figure S4). In contrast, all residues in FF3-60 had less probability of forming a helical structure than those in FF3-71 and the whole native helix formation probability was decreased to 77%. H3 had 10% chance to completely disappear.

H3 was a 3_10_ helix and had less stability than α-helix. Some termini residues of H1 and H2 had low probability of forming helical structures in FF3-71, which was consistent with the results of NMR experiments that the WT FF domain was conformational labile [5]. For FF3-71, we also compared 72 HN-O and N-O distances and 1757 hydrogen atom distances with experimental data and found that the positive violations consisted mainly of residues located in the *C*-terminal end of H2, H2-H3 loop and helix H3 (Supplement Figure S5 and S6). Compared with FF3-71, the helical structure formation became worse in FF3-60, especially for H3, and some termini residues of H1 and H2 were despiralized during the simulations in FF3-60. The conclusion could be obtained that the deletion of *C*-terminal helix affected the stability of the whole H3 and the terminal residues of H1 and H2.

### 2.3. Native Contact Formation

**Figure 4:**
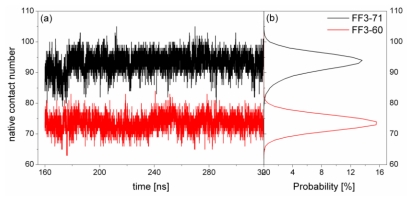
The native contact formation (residues 13–57) as a function of time (left panel) and distribution (right panel) during the simulations for native domain FF3-71 (black line) and truncated version FF3-60 (red line).

To test the changes in interactions between residues on account of deletion of the *C*-terminal helix, we calculated the native contact formation of FF3-71 and FF3-60 during the simulations. We defined a contact as being present if the Cα atom of two residues (*i*, *j* and |*i*−*j*| > 2) was within 8.5 Å [24]. Native contact was defined including all contacts which presented in 90% of conformational ensemble obtained from PDB. There were 117 native contacts obtained from residue T13 to E57 for the native state. The formations of native contact were plotted in Figure 4 for the two systems as a function of simulation time, together with their distributions. The results showed that there were only 94 native contacts being mostly formed during the simulations for the native version FF3-71 and the maximal contact number was 102. As discussed above, the FF domain might not be very stable. On the other hand, the MD method used currently required continuing refinement to reproduce the experiment results. Compared with the FF3-71, FF3-60 had much more native contacts unformed during the simulations and the native contact number being mostly formed was only 74, which was much smaller than that of the FF3-71. The deletion of the *C*-terminal helix changed the interactions among residues and some native interactions disappeared, which resulted in the instability of FF domain.

### 2.4. The Radius of Gyration

**Figure 5:**
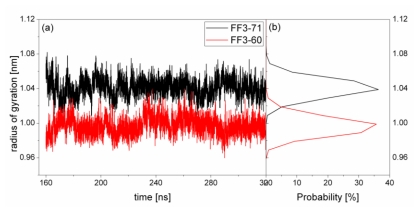
The radius of gyration (residues 13–57) as a function of time (left panel) and distribution (right panel) during the simulations for native domain FF3-71 (black line) and truncated version FF3-60 (red line).

The structural transformation sometimes leads to the change of compactness of structure. For a rough measure of the compactness of the structure after deletion of the *C*-terminal helix, the Rg was calculated and highlighted in Figure 5 together with the distribution. For FF3-71, the Rg changed from 1.01 nm to 1.09 nm and the value 1.04 nm had a maximal probability of 36%. For FF3-60, the Rg decreased and changed from 0.96 nm to 1.03 nm, and the value 1.0 nm had a maximal probability of 35.7%. The results meant that the FF3-60 was somewhat more compact than FF3-71. Namely, the truncation of *C*-terminal helix induced the compactness of the domain. Some non-native interactions might appear in the truncated version. In the intermediate state, some non-native interactions were detected at the interface of H2 and H3 [17].

### 2.5. H1-H3 Interface Interactions

**Figure 6:**
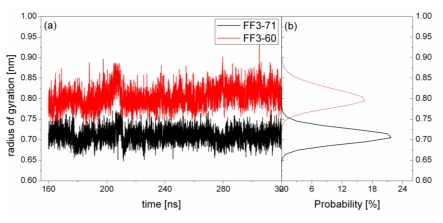
The radius of gyration (residues A17, A20, L24 L52 and L55) as a function of time (left panel) and distribution (right panel) during the simulations for native domain FF3-71(black line) and truncated version FF3-60 (red line).

**Figure 7:**
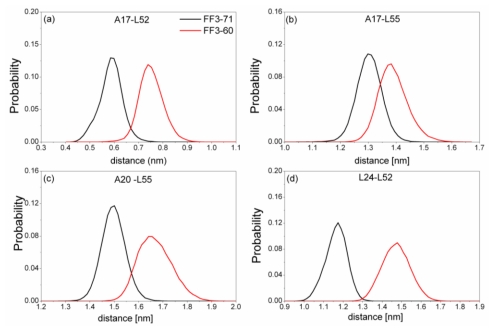
The distribution of distance between residues A17 and L52 (**a**), A17 and L55 (**b**), A20 and L55 (**c**), L24 and L52 (**d**) in FF3-71 (black line) and FF3-60 (red line).

The previous study indicated that the interactions formed at the H1-H3 interface comprising residues A17, A20, L24 of H1, and L52, L55 of H3 in the intermediate state were obviously different from those in the native protein [17]. To test the effect of the *C*-terminal helix on the H1-H3 interface interactions, we calculated the Rg of the interface residues mentioned above and its distribution for FF3-71 and FF3-60 (Figure 6). We also calculated the distances among the residues (Figure 7). The distance between two residues was defined as the distance between the two corresponding Cα atoms.

For FF3-71, the Rg of interface residues were changed from 0.65 nm to 0.77 nm and 0.71 nm had the maximal probability of 22%. By comparison, for FF3-60, the Rg of the interface residues were changed from 0.74 nm to 0.88 nm and 0.8 nm had the maximal probability of 16%. The region comprising interface residues became less compact in FF3-60 than that in FF3-71.

Totally, the distance between the interface residues in FF3-60 was larger than those in FF3-71 (Figure 7). The value with maximal probability was taken into account, from FF3-71 to FF3-60, the distance of A17-L52 changed from 0.59 nm to 0.74 nm, the distance of A17-L55 changed from 1.3 nm to 1.38 nm, the distance of A20-L55 changed from 1.5 nm to 1.65 nm and the distance of L24-L52 changed from 1.17 nm to 1.48 nm. The longer distance meant the weaker interaction. The results indicated that the interactions between H1-H3 interface residues were weakened owing to the deletion of *C*-terminal helix, which was consistent with the study on transition state [16].

Further studies of the conformations indicated that the position of H3 in relation to H1 changed, which induced the increases of the Rg and distances mentioned above. In addition, the despiralization of residue L55 (discussed in a later section), could also induce the augmentation of Rg of H1-H3 interface residue.

### 2.6. H2-H3 Interface Interaction

**Figure 8:**
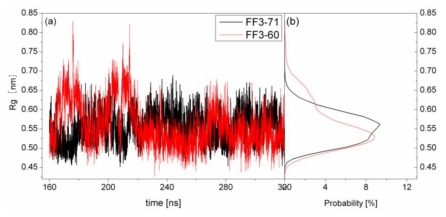
Time evolution (left panel) and distribution (right panel) of radius of gyration of residues A53, Y49, and I44 in the simulations in FF3-71 (black line) and FF3-60 (red line).

**Figure 9:**
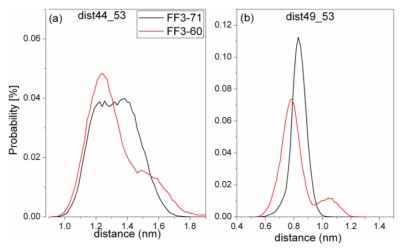
The distribution of distance between residues I44 and A53 (**a**), Y49 and A53 (**b**) in FF3-71 (black line) and FF3-60 (red line).

For truncated FF3-60, NMR experiments indicated that some non-native interactions in the intermediate state were observed at the H2-H3 interface involving a hydrophobic cluster consisting of A53, Y49, and I44 [17]. To confirm whether the deletion of *C*-terminal helix brought about these non-native interactions, we calculated the Rg of residues A53, Y49, and I44 and its distribution (Figure 8) and the distances of I44-A53 and Y49-A53 (Figure 9).

To take the value with the maximal probability into account, the Rg of interface residues was 0.56 nm for FF3-71 and 0.53nm for FF3-60, respectively. The reduction of Rg meant that the region comprising interface residues became compact with the deletion of *C*-terminal helix. The distance distribution curves of I44-A53 and Y49-A53 shifted to right along distance-axis. The interactions among those residues became stronger because of the deletion of *C*-terminal helix and some non-native interactions might have been caused. We could hypothesize that the deletion of the *C*-terminal helix might have driven the FF domain to the intermediate state.

With the deletion of *C*-terminal helix, the Rg of H2-H3 interface was decreased, but the Rg of H1-H3 interface was increased. That is to say, the deletion had a different effect on different sub-structures. Without the *C*-terminal helix, the position change of H3 in relation to H1 was detected and the angle made by H2 and H3 was varied (Figure S3).

### 2.7. The Spiralization and Despiralization for Some Residues

In intermediate state, H3 was elongated and L52 and A53, located in the turn between H3 and H4, joined H3 to form α-helix. At the same time, L55 and S56, located in the head of H4, were despiralized and formed a new turn [17]. We calculated the probability of forming helix for L52, A53, L55 and S56 during the simulations (Table 1) to confirm whether the truncated version FF3-60 would change towards the intermediate state or not. The data in Table 1 displayed that L55 and S56 were more stable in helix state in FF3-71 with probability of 99.9% and 100%, respectively. In FF3-60, the two residues had obvious despiralization tendency and the spiralization probabilities decreased to 52.1% and 58.9%. The probabilities of forming helix of L52 and A53 were 1.74% and 0.05% in FF3-60, which could not conclude that the two residues had a tendency to form helices because of the small value. However, compared with the probability of 0.05% and 0.0 in FF3-71, it was the fact that the two residues had more chance to form helical structures without the *C*-terminal helix.

The despiralization of L55 and S56 meant that some characters of the intermediate state were likely to come into being in FF3-60, and the structural transformation of FF3-60 had preference towards intermediate state.

## 3. Methods

The FF domain from human HYPA/FBP11 [15] (PDB entry 1UZC, includes residues 3 to 71, referred to as FF3-71) and the truncated version (includes residues 3 to 60, referred to as FF3-60) were chosen to be studied. The truncated version was derived from FF3-71 with the *C*-terminal helix (residues 61 to 71) being removed. MD simulations in the NPT ensemble were performed using the GROMACS software package [25,26] and GROMOS 43A1 [27] force field with simple point charge (SPC) water model [28].

Protonation states of ionizable groups were chosen for pH 7.0 and the *N*- and *C*-terminals were amidated and acetylated, respectively. The lysine and arginine residues side chain were protonated. The systems were solvated in a rectangular box with the minimum solute-box boundary distance being set to 1.4 nm. There were 7862 and 5232 SPC water molecules for FF3-71 and FF3-60, respectively. The system was first subjected to 1000 steps of steepest descents energy minimization. To maintain neutral condition, some counter-ions were added into the two systems (7 Cl− ions for FF3-71 and 6 Cl− ions for FF3-60) to replace the same number water molecules which had the highest or lowest Coulomb potential. Another energy minimization with the steepest descents method was performed to relax the ions and water molecules. To avoid unnecessary distortion of the protein when the MD simulation was started, a 500 ps equilibration run was first performed, in which the positional restraints were imposed on the solute atoms. During the equilibration run, the temperature was raised from 50 to 300 K. The initial velocities were taken from the Maxwell-Boltzmann distribution at 50 K. At last, continue MD simulations were performed without any restraints at 300 K for long time. All simulations were carried out at 1 atm. The neighbor list was updated every 10 steps and the periodic boundary conditions in all directions were adopted. The cut-off distance for the short-range neighbor list was 0.9 nm. The particle-mesh Ewald method was used to treat the long-range electrostatic interaction with a grid spacing of 0.12 nm and a fourth order interpolation [29,30]. The temperature and pressure of the system was kept constant by the Berendsen thermostat (coupling time 0.1 ps) and barostat (coupling time 0.1 ps) [31]. The time step for the MD integrator was set to 2 fs and SHAKE method [32] was applied to constrain all bond lengths with a relative tolerance of 10^−4^. The two independent simulations were lasted 320 ns for each system. The conformations were output every 2 ps and the last 160 ns-long trajectories were selected for the following analysis.

In the native state, there is a flexible *N*-terminal without any local regular secondary structure and the first helix starts from residue K14. For the convenience of comparison and the avoidance of the edge effects of truncating *C*-terminal, the segment consisting residues T13 to E57 were taken into account in the studies.

## 4. Conclusions

FF domain from human HYPA/FBP11 is a 71-residue four-helix bundle domain. In this study, we focused on the effect of the *C*-terminal helix on the stability of FF domain; the native state FF3-71 and its truncated version FF3-60 were studied by molecular dynamics simulations. The results indicated that the truncated version FF3-60 had obvious difference from the native partner FF3-71. The three helices H1–H3 exhibited instability and native contact number being formed during the simulation decreased obviously due to the deletion of *C*-terminal helix. Compared with FF3-71, the truncated version FF3-60 was more compact and had larger RMSDCα. This indicated that FF domain became unstable with the deletion of *C*-terminal helix. At the same time, without the *C*-terminal helix, the H1-H3 interface interactions became weak and some non-native interactions had the tendency of coming into being at H2-H3 interface, and the residues L55 and S56 presented obvious despiralization. These characteristics were similar to those in intermediate state. That is, the *C*-terminal helix played a crucial role in the stability of the FF domain [15,16].

Although it is improper to say the last 160 ns-trajectory of FF3-60 represents the intermediate ensemble of FF domain, it is a fact that the FF domain showed an obvious orientation towards the intermediate state owing to the deletion of *C*-terminal helix. With the elongation of the simulation time and more effective sampling methods, the intermediate state might be obtained by simulation of truncated version FF3-60.

## Supplementary Information



## Figures and Tables

**Table 1:** The spiralization probability for L52, A53, L55 and S56.

System	F_L52_ (%)	F_A53_ (%)	F_L55_ (%)	F_S56_ (%)
FF3-71	0.05	0.0	99.9	100
FF3-60	1.74	0.07	52.1	58.9
