# Supplementary material for: The Effect of C-Terminal Helix on the Stability of FF Domain Studied by Molecular Dynamics Simulation
Source: Int J Mol Sci. 2012 Feb 7;13(2):1720–32. doi: 10.3390/ijms13021720 (PMC3291988; doi:10.3390/ijms13021720)
Supplement: Supplementary file 1 [file ijms-13-01720-s001.pdf]

Supplementary Information

Figure S1. Cumulative number of conformational clusters that make up 95% of the trajectory in the simulations as a function of time. (**a**) FF3-71, (**b**) FF3-60. The RMSD of backbone atoms was used to cluster the conformation with cutoff of 0.09 nm for FF3-71 and 0.1 nm for FF3-60.

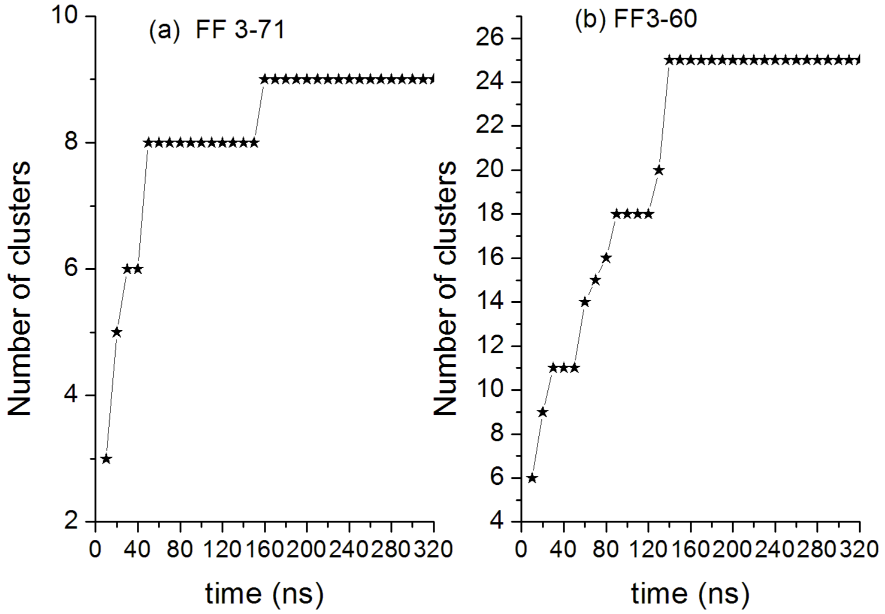

Figure S2. The distribution of radius of gyration for (**a**) FF3-71 and (**b**) FF3-60. The last 160-ns trajectories were divided into four parts depending on the simulation time and the radius of gyration of residues T13 to E57 were calculated for each part. The distribution of radius of gyration was the average of the four parts and the deviation labeled on the figure was the mean square error. The small deviation indicates the convergence of the simulations.

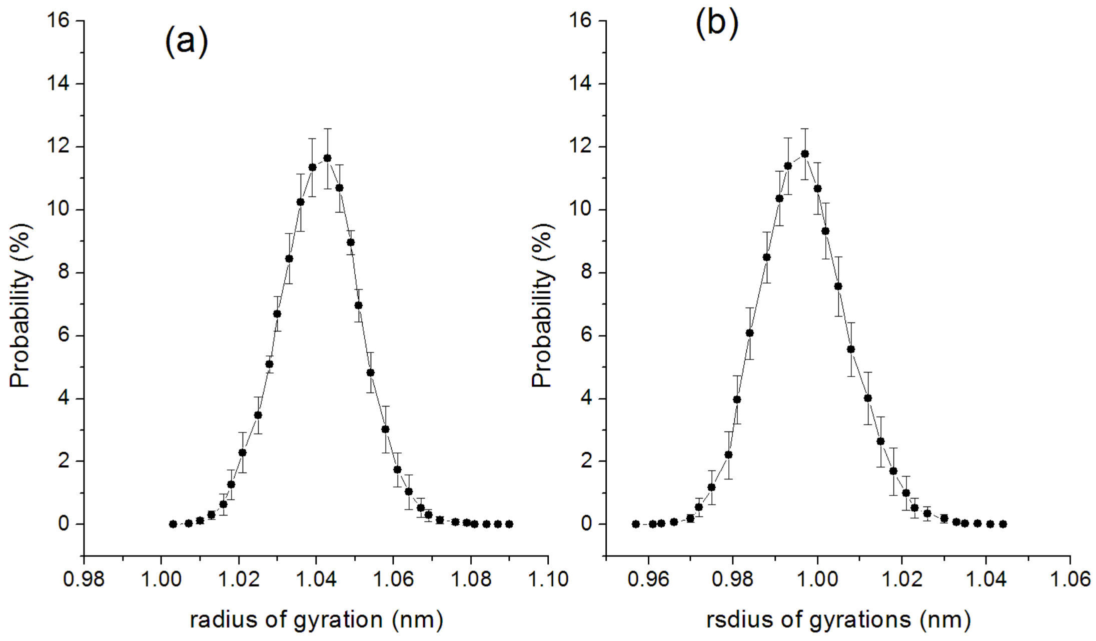

Figure S3. The central structures of major conformational clusters of two proteins. The green one is from FF3-71 and the red one is from FF3-60. For the convenience of comparison, only the residues 13-57 are taken into account. The RMSD of Cα atom of the two structures is 0.34 nm. Compared with that of FF3-71, the central structure of FF3-60 had the different orientation of H1. In the central structure of FF3-60, the position of H2 relative to H3 was changed, residue 51 was spiral and the length of H3 was longer than that in FF3-71, the residue 36 was unwound.

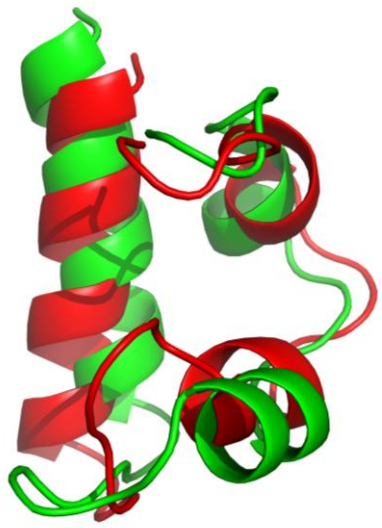

Figure S4. The helical formation of FF3-71 (■) and FF3-60 (●). H1, H2 and H3 were plotted in (**a**), (**b**) and (**c**). The helix classification was confirmed by program STRIDE. The length of helix was the number of residue forming helical structure determined by program STRIDE. For instance, the N-residue helix meant there were only *N* residues forming the helical structures and the other residues did not form helical structures in a certain region. In the native state, the lengths of helix are 15, 10 and 5 for H1, H2 and H3, respectively.

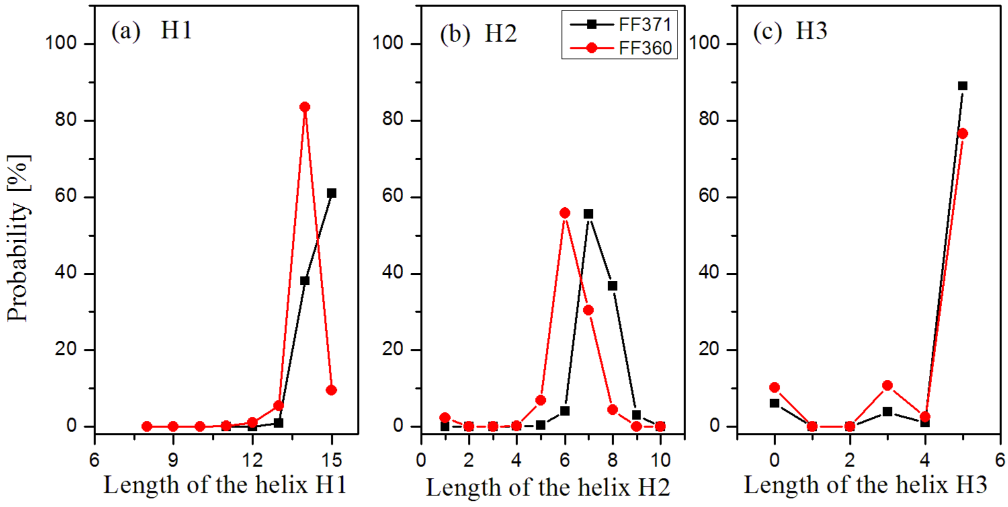

Figure S5. Distance bound violations in FF3-17. The experimental values of the 72 distance were used to restrict hydrogen bounds when the native structures were confirmed by NMR experiments [1]. The computational values of those distance are obtained from simulations trajectories by $<r^{-6}>^{-1/6}$. The positive or minus violation meant the calculated distance was larger or smaller than upper bound of corresponding experimental distance. There were small portion of distances with positive violation, which mainly related to residues located in the *C*-terminal end of H2, H2-H3 loop and helix H3 and corresponding hydrogen bonds were broken during the simulations. It was coincident with the results of helical formation analysis in text.

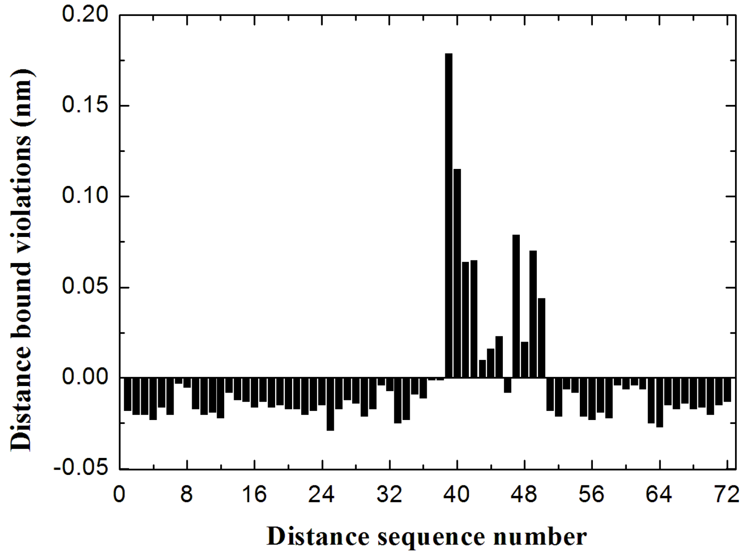

Figure S6. NOE distance bound violations in FF3-71. The 1757 NOESY distance bounds [1] were obtained from online database *NMR Restraints Grid* and The computational values of those distance are obtained from simulations trajectories by $<r^{-6}>^{-1/6}$. About 4.75% distances had positive violations and most computational values were in agreement with primary experimental data.

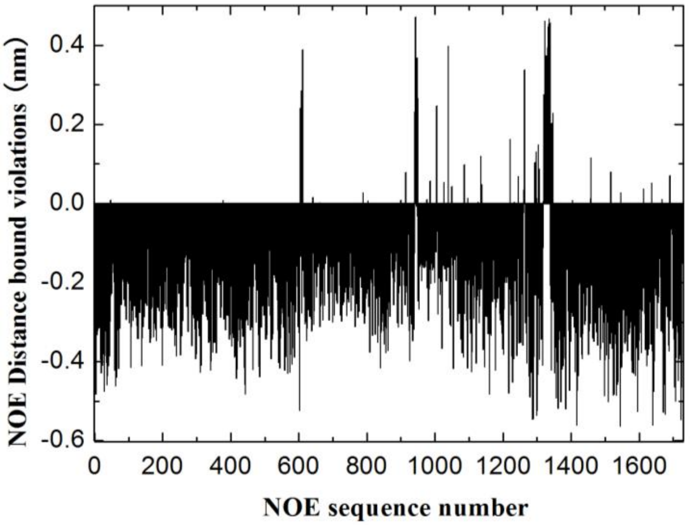

Reference

1. Allen, M.; Friedler, A.; Schon, O.; Bycroft, M. The structure of an FF domain from human HYPA/FBP11. *J. Mol. Biol.* **2002**, *323*, 411–416.

© 2012 by the authors; licensee MDPI, Basel, Switzerland. This article is an open access article distributed under the terms and conditions of the Creative Commons Attribution license (http://creativecommons.org/licenses/by/3.0/).
